# Long-term endoscopic submucosal dissection with postoperative lung injury: a case report

**DOI:** 10.1186/s12893-021-01440-8

**Published:** 2021-12-27

**Authors:** Qian-Mei Zhu, Hong Tu, Bing Hu, Xiao Wang

**Affiliations:** 1 Present Address: Department of Anesthesiology, West China Hospital, Sichuan University & The Research Units of West China (2018RU012), Chinese Academy of Medical Sciences, No. 37 Guoxuexiang, Wuhou District, Chengdu, 610041 Sichuan China; 2 Department of Anesthesiology, National Cancer Center/National Clinical Research Center for Cancer/Cancer Hospital, Chinese Academy of Medical Sciences and Peking Union Medical College, No. 17 Panjiayuan Nanli, Chaoyang District, 100021 Beijing, China; 3 Department of Gastroenterology, West China Hospital, Sichuan University & The Research Units of West China (2018RU012), Chinese Academy of Medical Sciences, No. 37 Guoxuexiang, Wuhou District, 610041 Chengdu, Sichuan China

**Keywords:** ESD, ALI, Long-term, Perforation, Case report

## Abstract

**Background:**

Endoscopic submucosal dissection (ESD) has been recognized as a safe and minimally invasive technique for the removal of early gastric cancer. Here, we describe a case of extended-duration ESD for a gastric tumor associated with intraoperative perforation and bleeding. Unfortunately, the patient developed acute lung injury (ALI) after the operation.

**Case presentation:**

A 72-year-old woman received ESD for a gastric tumor under general anesthesia. Preoperatively, endoscopic ultrasonography (EUS) showed a 3.1 × 3.5 cm hypoechoic, well-defined mass at the junction of the antrum and body of the stomach on the greater curvature, originating in the muscularis propria layer. During the ESD procedure, when the submucosal mass was stripped, it was found to be closely adhered to the muscular layer and serosa layer, and a full-thickness incision was performed. The abdominal cavity was gradually filled with carbon dioxide gas, and abdominal puncture was performed to reduce intra-abdominal hypertension (IAH). Because the mass adhered to the greater omentum and there was more bleeding during the operation, a long duration of hemostasis and suturing of the wound was required. The whole operation lasted nearly 9 h, and total blood loss was 800 ml. After surgery, acute lung injury was suspected, and the patient was sent to the intensive care unit (ICU) for further treatment.

**Conclusions:**

The operation time of ESD and IAH caused by perforation are closely related to a poor prognosis. We should pay attention to the impact of operation time on patients and improve awareness regarding protecting important organ functions.

## Background

Endoscopic submucosal dissection (ESD) is a well-practiced and safe minimally invasive technique for the removal of early cancers and large lesions from the luminal gastrointestinal tract [1]. However, perforation and bleeding are two major and serious ESD-related complications [2]. Studies have shown that when the diameter of gastric tumors is larger than 3 cm, the intraoperative perforation rate can be as high as 12%, and the bleeding rate can be as high as 6% [3]. We report a case of an older woman who underwent a 9-h procedure due to bleeding and perforation during ESD for gastric tumor. Unfortunately, the patient developed acute lung injury (ALI) after the procedure. Informed consent for publication was obtained from the patient.

## Case presentation

A relatively healthy 72-year-old woman, weighing 49 kg and with a height of 143 cm, received ESD for a gastric tumor under general anesthesia. She had only a history of hypertension and took medication regularly. Preoperative examinations, including white blood cell count, platelet count, coagulation system and electrocardiogram (ECG), were all normal. Endoscopic ultrasonography (EUS) showed a 3.1 × 3.5 cm hypoechoic, well-defined mass at the junction of the antrum and body of the stomach on the greater curvature, originating in the muscularis propria layer. The pathology of biopsy specimens included a large number of red blood cells, a small number of spindle cells and proliferative epithelial cells, which did not rule out the possibility of neurogenic cells; severe chronic inflammation of the mucosa with a lymphoproliferative aspect was also detected. After discussion with the patient and her relatives, endoscopic resection was conducted, and written informed consent was obtained from the patient before the operation.

**Fig. 1:**
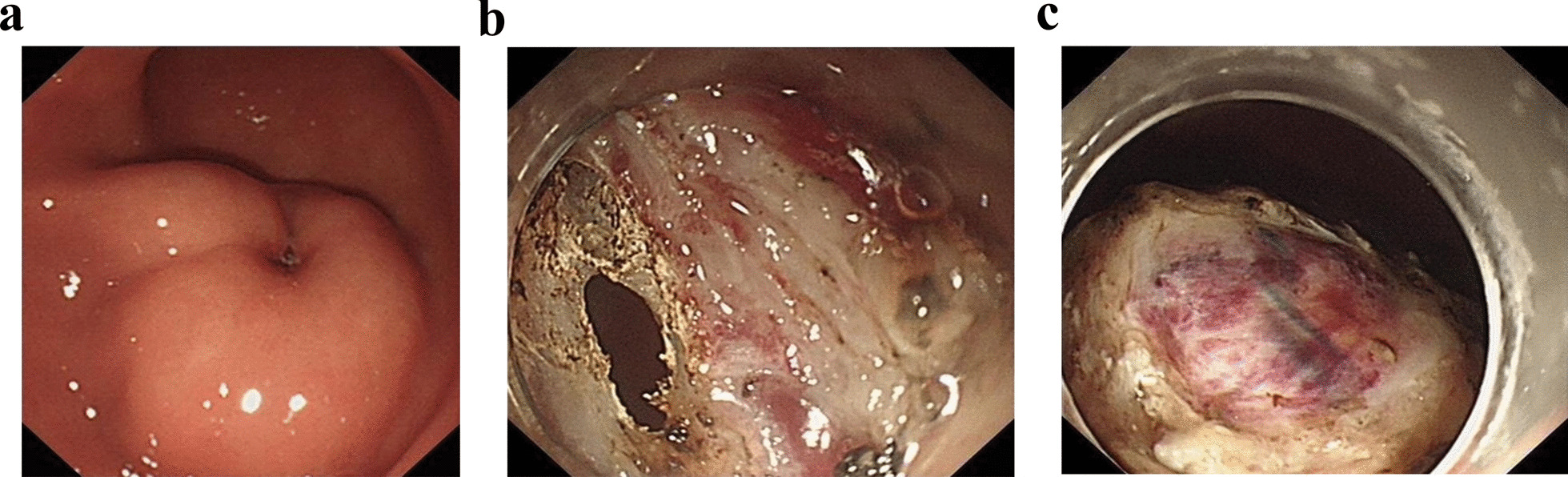
Gastroscopy images in the surgery. **a** Before surgery, a 3.1 × 3.5 cm hypoechoic mass at the junction of antrum and body of stomach was seen. The center of the tumor was sunken and tough. **b** Intraoperative perforation. **c** The perforation was sutured at the end of the surgery

Before the procedure, the patient’s vital signs were normal. Mechanical ventilation (volume controlled and FiO_2_ was 50%) was performed after induction of general anesthesia; anesthesia was maintained with propofol and remifentanil. Based on gastroscopy, there was a protuberant lesion with a size of approximately 3.1 × 3.5 cm, with a central depression and soft touch (Fig. 1a). A lesion mass was seen after mucosal incision with a dual knife. When the submucosal mass was stripped, it was found to be closely adhered to the muscular layer and serosa layer, and a full-thickness incision was performed. The gastric perforation is shown in Fig. 1b. The abdominal cavity was gradually filled with carbon dioxide gas, and the peak airway pressure gradually increased to 30–35 cm H_2_O. Another surgeon performed abdominal puncture to reduce the high intra-abdominal pressure. The mass adhered to the greater omentum, and there was more bleeding during the operation. First, foreign body forceps and hot biopsy forceps were used for hemostasis. After the bleeding stopped, the lesion was completely removed. The wound was sutured with a titanium clip and nylon ring double-layer purse (Fig. 1c). The whole operation lasted nearly 9 h, and total blood loss was 800 ml.

**Fig. 2:**
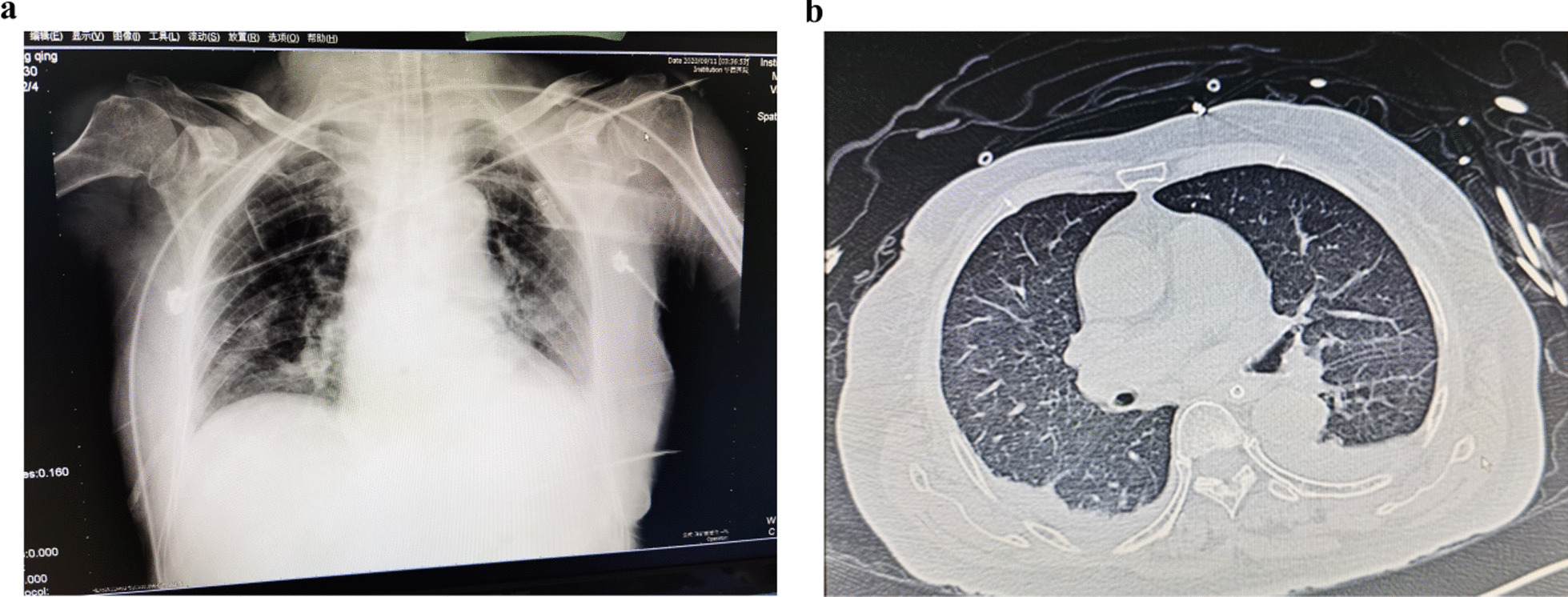
Chest imaging examinations. **a** Chest X-ray on the day after surgery showed scattered inflammation in both lower lungs. **b** Computed tomography (CT) on the 4th day after surgery demonstrated scattered inflammation in both lungs

During recovery in the postanesthesia care unit (PACU), the patient was found to have a fast respiratory rate (40–45 bpm) and low tidal volume (120–170 ml), even after intravenous administration of neostigmine and atropine to recover breathing. PetCO_2_ was 45–55 mmHg and SpO_2_ 100%. Venous blood gas analysis showed that the pH was 7.24 and PCO_2_ 64.3 mmHg. From lung auscultation, the breath sounds of both lungs were thick, and scattered moist rales were heard. Considering that the operation time was too long, long-term intra-abdominal hypertension (IAH) and mechanical ventilation had caused obvious damage to the patient’s lung function, she was transferred to the intensive care unit (ICU) with an endotracheal tube for further improvement. Bedside chest X-ray on the day after surgery revealed scattered inflammation in the lung (Fig. 2a). The patient was diagnosed with pulmonary infection and acute respiratory distress syndrome (ARDS). Four days later, chest computed tomography (CT) still showed scattered inflammation in both lungs, especially in the lower lobes, and a small amount of pleural effusion was found on both sides (Fig. 2b). After 7 days of anti-inflammatory and gastric protective treatment in the ICU, the tracheal tube was removed, and the patient transferred back to the general ward on the 8th day.

## Discussion and conclusions

It has been reported that most ESD procedure times are no more than 2 h [4], and such long-duration ESD for gastric tumors has not been reported to date. There were unexpected difficulties during this procedure: as the mass had invaded the muscular layer and serosa layer, a full-thickness incision was necessary to completely cut off the tumor. In addition, endoscopic hemostasis and tumor stripping are more difficult and time-consuming than an open operation, which leads to such a long procedure time. Prolonged mechanical ventilation, IAH caused by gastric perforation and possible intraperitoneal infection resulted in obvious ALI and a prolonged postoperative hospital stay. Endoscopic surgery is difficult, and open surgery should be considered if the perforation cannot be closed in a timely manner.

Because endoscopic management can be equal to or even superior to conventional surgery in terms of long-term survival [5, 6], the number of patients undergoing ESD is increasing. It is undeniable that minimally invasive treatment is more likely with elderly patients. As the recently published guidelines describe [1], the absolute indications for ESD are “(i) nonulcerated, clinically intramucosal (cT1a), differentiated-type carcinomas with a long diameter > 2 cm; (ii) ulcerated, cT1a differentiated-type carcinomas with a long diameter ≤ 3 cm; and (iii) nonulcerated, cT1a undifferentiated-type carcinomas with a long diameter ≤ 2 cm”. Expanded indications are defined as lesions that are presumed to have a < 1% risk of lymph node metastasis. Relative indications are those who do not meet the requirements for absolute or expanded indications, or open surgery cannot be recommended when taking into account the patient’s condition. Cases with gastrointestinal tumors are prone to intraoperative or postoperative perforation if the tumor originates from the deep muscularis propria layer or the muscle layer is damaged during ESD treatment, which has been described in many reports [3, 7, 8]. Similarly, endoscopic treatment of diverticulum-associated intestinal tumors is challenging due to the lack of a muscle layer [9]. However, there are cases of esophageal schwannoma involving the muscle layer without serious complications after ESD treatment [10].

In general, progress in surgical and related anesthesia technology has a goal of meeting the needs of patients. Endoscopic minimally invasive technology for the treatment of early gastrointestinal cancer is also based on patient benefit. However, regardless of the surgical method selected, the operation time should be strictly controlled. An operation time ≤ 1 h was once recommended to reduce the risk of postoperative infection in retrograde intrarenal surgery [11]. Ziolkowski et al. also reported that burn surgery for more than 3 h will increase postoperative complications [12]. If it is expected that the operation is difficult and that the duration would be long, whether to change the operation mode should be considered by weighing the benefit to the patient. Although technology needs breakthroughs, surgeons must seriously consider the benefits of surgical decision-making for individual patients. Anesthesiologists should also make efforts to protect organs systems, especially the lung.

In addition to intraoperative perforation and a large amount of total blood loss, the manifestation of postoperative ALI was a major concern in this case. The patient had no history of acute pulmonary inflammation or any chronic pulmonary disease before surgery. However, signs of pulmonary infection appeared from auscultation, as confirmed by postoperative chest X-ray and CT. The incidence of postoperative pulmonary complications in patients receiving ESD under general anesthesia is very low when compared with conscious sedation [13, 14], and postoperative pneumonia is often associated with intraoperative reflux aspiration [15]. In this case, we considered that the main factors of her ALI included long-term mechanical ventilation, systemic inflammatory response caused by gastric perforation, long-term IAH and advanced age. The long-term increase in intra-abdominal pressure (IAP) leads to increased airway pressure, decreased lung compliance, decreased diaphragm activity, atelectasis and hypercapnia. On the other hand, IAH can lead to insufficient perfusion of organs and aggravate multiple organ injury [16]. Gastric perforation itself can cause leukocyte activation, chemotaxis, leukocyte adhesion and vascular instability, which may result in ARDS [17].

Herein, we present a case of a long-duration ESD procedure for treating early gastric cancer. Based on this case, we realize that the operation time for ESD and IAH caused by abdominal organ perforation were closely related to the poor prognosis of patients. This case does not refute the advantages of ESD in benefiting patients; however, when treating patients with different conditions, the decision-making ability of endoscopists and anesthesiologists should be strengthened to reduce the incidence of postoperative complications.

## Data Availability

Not applicable.

## Abbreviations

ESD: Endoscopic submucosal dissection
ALI: Acute lung injury
EUS: Endoscopic ultrasonography
ECG: Electrocardiogram
PACU: Post anesthesia care unit
CT: Computed tomography
IAH: Intra-abdominal hypertension
ICU: Intensive care unit
SpO_2_: Pulse oxygen saturation
ARDS: Acute respiratory distress syndrome
IAP: Intra-abdominal pressure
